# Artificial social intelligence in teamwork: how team traits influence human-AI dynamics in complex tasks

**DOI:** 10.3389/frobt.2025.1487883

**Published:** 2025-02-17

**Authors:** Rhyse Bendell, Jessica Williams, Stephen M. Fiore, Florian Jentsch

**Affiliations:** ^1^ Cognitive Sciences Laboratory, Institute for Simulation and Training, University of Central Florida, Orlando, FL, United States; ^2^ Department of Philosophy, University of Central Florida, Orlando, FL, United States; ^3^ Team Performance Laboratory, Institute for Simulation and Training, University of Central Florida, Orlando, FL, United States; ^4^ Department of Psychology, University of Central Florida, Orlando, FL, United States

**Keywords:** human-agent teams, artificial social intelligence, theory of mind, team cognition, team process, team performance

## Abstract

This study examines the integration of Artificial Social Intelligence (ASI) into human teams, focusing on how ASI can enhance teamwork processes in complex tasks. Teams of three participants collaborated with ASI advisors designed to exhibit Artificial Theory of Mind (AToM) while engaged in an interdependent task. A profiling model was used to categorize teams based on their taskwork and teamwork potential and study how these influenced perceptions of team processes and ASI advisors. Results indicated that teams with higher taskwork or teamwork potential had more positive perceptions of their team processes, with those high in both dimensions showing the most favorable views. However, team performance significantly mediated these perceptions, suggesting that objective outcomes strongly influence subjective impressions of teammates. Notably, perceptions of the ASI advisors were not significantly affected by team performance but were positively correlated with higher taskwork and teamwork potential. The study highlights the need for ASI systems to be adaptable and responsive to the specific traits of human teams to be perceived as effective teammates.

## 1 Introduction

Teams are employed to help address dynamic and complex objectives that would be too complicated, intricate, multidisciplinary, or large in scope for individuals or uncoordinated groups to solve on their own (Fiore et al., 2010; Kilcullen et al., 2023; Lyons et al., 2021). This is often for situations such as when quick decisions are necessary and errors can have severe consequences, for task environments that are ambiguous, stressful or ill-defined, and, especially, for when the lives of others depend on the collective knowledge and insights of a team, such as in military, healthcare and aviation (Salas et al., 2008). Generally, teams are considered to involve two or more members working interdependently toward a common goal through either face-to-face or virtual interaction, where members have different roles and responsibilities (Kozlowski and Bell, 2013; Mathieu et al., 2014; Salas et al., 1992). Teams are often interdisciplinary in nature, which help them achieve emergent outcomes with only partially overlapping knowledge; here, members maintain enough of an understanding of each other’s competencies or disciplines to engage in teamwork and achieve team goals (Fiore, 2008).

This understanding, emerging across team members, may include knowledge of team member roles, individual abilities, resources available, task demands, team goals and requirements. This is generally referred to as mental models, which are the organized knowledge structures that allow one to predict and explain their environment, recognize and remember relationships among components of the environment, and help us to describe, explain, and predict events in the environment (Johnson-Laird, 1983). Team members having mutually held, though not necessarily identical, mental models, help them predict what their teammates will do, enabling them to adapt and respond to changing task demands (Cannon-Bowers et al., 1993; Mathieu et al., 2000). Involved in both developing and utilizing shared mental models, humans draw upon a socio-cognitive process that is generally referred to as Theory of Mind (ToM). This is a form of mental state attribution whereby we perceive and interpret behaviors and social cues (e.g., facial expressions) to infer intentions and knowledge of another to support social interactions. This helps us make informed predictions about future states and scenarios, facilitating cooperation and coordination in varied contexts (Baron-Cohen et al., 2001; Cuzzolin et al., 2020; Wellman, 2011; Williams et al., 2022). At a more general level, ToM is a fundamental part of social intelligence, playing a large role in social interaction, collaboration and communication (Zhang et al., 2012). ToM is critical to a team’s ability to develop, retain, and update shared mental models concerning the task, the resources available, team interaction patterns, and different team members knowledge, skills and abilities. Members utilize ToM to anticipate needs, actions, and future problems within the team (Demir et al., 2017; Chen and Barnes, 2014). In short, the effective use of ToM, along with development of shared mental models, enable high-performing human teams to coordinate behavior and knowledge in multiple ways. This includes, but is not limited to, arriving at common interpretations of dynamic contexts, such that they are in sync with the task situation, inferring what courses of action to take considering the context, identifying what information is important to share, and determining the most effective timing for when to share that information; these features are often observed in high-performing and effective teams (Cannon-Bowers et al., 1993; Lyons et al., 2021; Salas and Fiore, 2004; Tannenbaum and Salas, 2020).

Within the general study of teams, research has examined many factors that impact processes and performance (O'Neill et al., 2023), including factors that are more taskwork or teamwork oriented. For example, teamwork-related factors include general team competencies. This includes generic skills that all team members need regardless of the task context, such as communication skills, the ability to develop and maintain shared mental models (Mathieu et al., 2000), as well as specific team competencies that are relevant to a particular time situation and are more related to the individuals, roles, and abilities held by team members (Bowers et al., 2000; Fiore, 2008). As a complement to this, taskwork reflects the components of teaming that largely demand independent actions or reasoning as opposed to interaction with other team members (Salas et al., 2008). Although, one could easily imagine situations where this could differ depending on a given task context, such as in construction where moving heavy resources into place could require more than one individual to carry and stabilize objects, tasks that are interdependent would necessarily involve teamwork to effectively coordinate abilities and actions across multiple individuals (Salas et al., 2008).

We introduce this teamwork *versus* taskwork distinction because Artificial Intelligence (AI) and automation have typically been utilized by humans teams in utilitarian ways (e.g., as a tool) to accomplish taskwork (Musick et al., 2021; Phillips et al., 2011). Further, they have been typically developed/employed as static, deterministic models and expert systems that operate through rules that have been pre-defined by a human (Kaplan and Haenlein, 2019). Although this form of advanced technology certainly augments teamwork, we submit that, for AI to operate effectively as teammates, AI agents must not simply execute tasks, they must also engage in social and interpersonal ways with their human teammates (Demir et al., 2023). Recent advancements in computer science have allowed AI to autonomously complete increasingly sophisticated and dynamical functions in both taskwork and, more recently, teamwork (Farah and Dorneich, 2024; Seeber et al., 2020; Zhang et al., 2023). These advances illustrate AI being increasingly utilized to augment and partner with human teams (Demir et al., 2017), as well as fulfill viable roles within human teams (McNeese et al., 2018; Sycara and Lewis, 2004). In the near future, AI will be as agentic as humans, with the ability to engage in decision-making, adaptation, and communication (O’Neill et al., 2022), such that these autonomous agents can be considered a teammate as opposed to a tool (McNeese et al., 2018). Teams where humans partner with an AI agent are typically referred to as Human-Autonomy Teams (HATs; McNeese et al., 2018), sometimes also referred to as a Human-Agent Team (same acronym, HAT), and are comprised of at least one human and at least one autonomous/artificially intelligent agent An autonomous agent can be considered a team member when it is able to fulfill a distinct role and make its own contributions to the team’s performance (O’Neill et al., 2022), and when the agent has some degree of decision-making freedom, as teammates typically have the capability to be proactive, choose or recommend courses of action (Wynne and Lyons, 2018).

This means that all members of HATs will need to be able to communicate and establish common ground among team members, including their understanding of their environment and tasks, their shared mental models, and shared team goals (Fiore et al., 2021; Lyons et al., 2021), necessarily requiring an AI team member, as part of the team, to possess social intelligence to meaningfully contribute to effective teamwork and collaboration (Li et al., 2022; Williams et al., 2022). Thus, an autonomous agent will need to be designed to incorporate some form of artificial social intelligence (ASI) to engage in meaningful human-agent collaboration. This will require an agent to possess some form of social competence, enabling it to engage in effective information and social exchanges supporting effective team processes (Best et al., 2016; Fiore et al., 2013; Wiltshire et al., 2014). As previously discussed, human teams use ToM processes, social intelligence, and their shared understanding and commonly held mental models to effectively engage in teamwork. We submit that AI teammates, will need to be capable of what we’ve termed Artificial Theory of Mind (AToM; Williams et al., 2022). The challenge is that agents lack the rich, embodied, social experiences that humans learn from to inform and build their ToM competence throughout development and the lifespan. As such, interdisciplinary research is needed to study how AI can develop AToM, whether it be learning through interaction or learning via observation. Regardless, such approaches require significant amounts of training data for ASI to be able to function in varied social and collaborative contexts (Hofstede, 2019; Williams et al., 2022).

To address the confounds introduced by complex, naturalistic scenarios, foundational research on artificial Theory of Mind (AToM) has relied on highly controlled problem spaces, such as token-based tasks and predefined scenarios (De Weerd et al., 2017; Ruocco, Mou, Cangelosi, Jay and Zanatto, 2021; Sun et al., 2022). These studies aim to isolate specific cognitive components, like belief formation and goal inference, offering valuable insights into the mechanics of ToM and the challenges involved in integrating it into virtual agents. Notably, however, these efforts have not yet produced conclusive evidence that AToM meaningfully enhances agent performance or interaction outcomes. Furthermore, the precise functions and characteristics of a useful AToM remain under exploration and may differ significantly from human theory of mind. While early studies provide valuable groundwork, they illustrate that artificial ToM may need to fulfill distinct roles in human-agent interaction to be effective.

Controlled experimental setups, such as those involving logic-based tasks (Goodie et al., 2012) or game-like structures (Meijering et al., 2011), demonstrate the utility of simplified tasks for investigating higher-order ToM. These studies show that agents can engage in strategic reasoning and recursive thinking under highly structured conditions, offering useful insights into ToM mechanisms. However, reliance on scaffolding in these setups such as breaking tasks into discrete, manageable steps limits their applicability to real-world settings. In practical team scenarios, agents must handle simultaneous, competing demands without explicit instructions or external guidance. Moreover, these studies often assume that cognitive strategies developed in structured contexts will transfer seamlessly to more open-ended interactions. But this assumption breaks down when agents need to adapt dynamically to unpredictable human behavior.

Efforts to test ToM within negotiation tasks further illustrate both the promise and the limits of foundational studies. De Weerd et al. (2017) demonstrated that recursive reasoning enhances agent performance in negotiations by enabling agents to anticipate both cooperative and competitive actions. However, the fixed, turn-based nature of these scenarios constrains the flexibility required in real-world interactions, where goals and strategies are fluid. Comparisons between studies like De Weerd et al. (2017) and Sun et al. (2022) highlight the fact that although these approaches offer useful insights into AToM capabilities, they remain limited in their ability to generalize to naturalistic environments where tasks evolve unpredictably.

Research examining trust dynamics with virtual agents also underscores the importance of adaptive behavior. For example, while Ruocco et al. (2021) showed that basic ToM capabilities can foster initial trust, trust tends to plateau without richer, context-aware exchanges. This need for sustained engagement mirrors challenges observed in negotiation-based studies, where agents with higher-order reasoning excel in specific tasks but encounter limitations in more fluid, real-time interactions. Taken together, these findings emphasize the importance of adaptive ToM for maintaining effective collaboration across dynamic, multi-agent environments.

Studies on social intelligence and anthropomorphism add further complexity to the development of effective ToM agents. For instance, Sturgeon et al. (2021) found that predictable behavior influenced participants’ perceptions of a robot’s cognitive abilities, complicating efforts to implement ToM intuitively. Similarly, van der Woerdt and Haselager (2019) demonstrated that attributions of agency and responsibility are shaped by a robot’s behavior and predictability, suggesting that rigid, pre-scripted ToM behaviors can diminish perceptions of social intelligence. These findings highlight the need for agents to demonstrate adaptive, nuanced behavior that aligns with real-world human expectations.

Our study builds on these foundational efforts by examining AToM within dynamic, high-stakes team tasks that reflect the unpredictability and interdependence typical of real-world human-agent interactions. Unlike simplified setups that limit agent adaptability, our approach requires agents not only to infer human goals and intentions but also to manage uncertainty, adapt to shifting conditions, and provide context-aware interventions. This shift toward more naturalistic environments highlights the need for agents capable of generating and maintaining meaningful beliefs in real time, which are abilities that are essential for fostering effective collaboration in complex, real-world scenarios.

A near-term, promising approach to developing AToM is through the use of profiling, which involves creating machine-readable, quantified descriptions of individual team members’ characteristics, which AI can then use to inform its internal models of humans (Bendell et al., 2024). These profiles provide theory-driven *a priori* data to agents in order to provide a basic initial model of their team members individual differences, capabilities, and roles that are reasonably expected to be relevant to the task. Research has shown that traits such as personality, social skills, and teamwork competencies significantly influence team dynamics, including communication, coordination, and overall performance (Larson and DeChurch, 2020; Morgeson, 2005; Weidmann and Deming, 2021; Van Eijndhoven et al., 2023). However, effective profiling requires capturing both a sufficient quantity of data, as well as situationally relevant information that allows AI to make accurate inferences about its teammates’ mental states and predict future actions (Williams et al., 2023a). Profiles can enable ASI agents to develop a functional understanding of human teammates, which is critical for social interactions and for contributing to the team beyond task execution. In this way, profiles can act as a surrogate for shared mental models in that they help an ASI understand more generic competencies as discussed in the general literature on teams (Bowers et al., 2000). Specifically, it is only through many interaction episodes that teams acquire shared understanding about the capabilities of their teammates. But team profiles can provide immediate insights into a human team member’s potential for effectiveness, thus accelerating the acquisition of background knowledge helpful for coordinating teamwork.

Moreover, how the ASI functions within a team, team member predispositions, agent interactions with team members, and a myriad of other factors can influence perceptions of the agent teammate (Bendell et al., 2023). Inappropriate interactions can disrupt team processes, including information exchange and coordination, leading to adverse teaming outcomes (Demir et al., 2016; Demir et al., 2018) Further, perceptions of the autonomous teammate can be influenced, impacting potential future interactions, reliance behaviors, and teaming success (Parasuraman and Riley, 1997), including the misuse or even disuse of the agent if their abilities and contributions are thought not to be of value (Talone, 2019).

This research was conducted as part of a larger program investigating artificial socially intelligent agents (DARPA, 2019; ASIST, 2023). Specifically, the work reported in this manuscript sought to examine the relationships between human team members and ASI advisors that collaborated to complete missions requiring teamwork. A key feature of the study is that the ASI advisors were “real” autonomous agents. Unlike the majority of human-AI teaming research thus far, which relies on “wizard of oz” manipulations with human confederates acting as AI, this study was designed to develop functioning AI architectures that leverage artificial theory of mind (AToM) and engage explicitly in teamwork with their human counterparts. Further, the team missions we examined represented actual interactions between human teammates and AI observing, processing, and engaging with the team in real time.

We aimed to answer several questions regarding how teams perceived themselves (e.g., their processes, satisfaction with outcomes, and team-level self-efficacy) as well as how they perceived their AI advisor. Building upon previous work, we employed a profiling model that allowed us to discriminate between teams that were relatively higher in potential for effective taskwork (Taskwork Potential) and for effective teamwork (Teamwork Potential). Examining teams through the lens of those profiles, we have shown that the measurable differences in teams’ compositions can be predictive of how positively teams view themselves and their AI advisors (Bendell et al., 2023). This paper continues that line of research. Specifically, although we found that teams that were higher in taskwork and teamwork potential were associated with more positive perceptions of both their team and team’s advisor, it is unclear whether those responses are better or more appropriately calibrated to the reality of their experience (Bendell et al., 2023). It is possible, and potentially problematic, that teams with higher tasking and teaming potential may provide more positive ratings even when their artificial advisors are not effective team members or when their teamwork as a whole breaks down.

## 2 Methods

The experiment from which our data are derived was conducted as part of the Artificial Social Intelligence Supporting Teams (ASIST) Program. The study was conducted to evaluate the effectiveness of Artificial Social Intelligence (ASI) in enhancing teamwork processes in *ad hoc* teams and performance in a simulated task environment (Huang et al., 2023). The focus was on measuring the ASI’s impact on team states such as motivation, processes like synchronization, and overall mission outcomes including game score. This study involved human participants (3 per team) collaborating with an ASI advisor that provided real-time, text-based advice and guidance to improve teamwork. Notably, this study featured bi-directional communication between humans and AI through a flexible prompt-reply system, which allowed participants to select from a set of response options after receiving a communication from their assigned artificial advisor. The data collected for this study as generated in a large-scale online public testbed, with automatically initiating servers that were able to instantiate new instances of the experimental testbed as needed, affording the simultaneous (e.g., multiple trials and teams going at once) and distributed (e.g., individuals are not co-located) data collection of 1,160 experimental trials. The data collected for this study are available as part of a publicly available dataset (Huang et al., 2024), which contains both the trial and simulation data, as well as access to the testbed-related files needed for other researchers who may want to use this testbed.

### 2.1 Experimental task

The experimental task was designed specifically to assess how well AI architectures focusing on artificial social intelligence could improve team outcomes. The task was structured to impose time constraints and ensure that teams were under a reasonably high demand when executing bomb disposals (e.g., execution of the taskwork elements). From this, the number and complexity of bombs placed in the simulated testbed environment was tuned to require teams to work efficiently and collaboratively to succeed. However, the taskwork components of the design were implemented explicitly to incorporate team member interdependencies because teamwork and the processes that teams execute to succeed at teaming were the primary interests of the research program. Some details of those interdependencies and the teamwork they elicited are discussed below; however, a full accounting of the task design is available alongside the publicly available dataset (Huang et al., 2024).

Regarding the technical constraints and design of the ASI advisors, some design decisions were necessary given the current state of tools for interfacing AI with teams that are operating in real time. One key design feature was the limitation of participant communications to a text chat interface and in-game annotation tools. The latter allowed participants to select from a subset of messages and broadcast that message from the location at which they used the tool. For example, participants could use the in-game annotation tools to request that teammates rally to them, ask their teammates to provide medical assistance or bring extra tools, to warn their team of fires, or to broadcast the locations of bombs and other hazards. Although those communication avenues present some limitations compared to voice communications, we note that they are common channels that can be effective for supporting teamwork especially in high demand virtual environments and they offer a viable solution while avoiding the need for online natural language processing. Notably, communication between the ASI advisors and human teammates was further constrained and structured to support meaningful exchanges but avoid many of the issues that can be caused by faulty or incomplete natural language processing. Specifically, ASI delivered advice and messages to team members in the form of texts that appeared on the members’ screens in association with response options prepared by the ASI. The human team members could not freely respond to their ASI counterparts, which was a limiting but important design choice made to ensure that the ASI could understand responses and continue structured and effective exchanges.

The following subsections provide an overview of the task structure, the implementation of the taskwork interdependencies, and the features and functions designed to facilitate or require teamwork behaviors.

### 2.2 Task structure

The simulated testbed environment (STE) constructed for ASIST Study four was built up from the java release of the popular sandbox MMO Minecraft (Mojang, 2015), and was designed to feature two distinct areas: one problem space, the “field”, in which participants searched for and disposed of bombs, and one planning space, the “store”, in which participants could execute a range of collaborative actions that would serve their teamwork processes in the field. When operating in the field, participants could freely control their Minecraft avatars and employ different tools to support their objectives. This included tools to help locate bombs, communicate and record those locations on a shared mini-map, dispose of those bombs by applying the correct tools in the correct order, and manage the spread of fires caused by the explosion of some particularly volatile bombs. A time limit of 10 min was imposed on teams’ field operations. Critically, teams could vote to leave the field and teleport to the store, which was a separate location in the Minecraft environment. As long as teams were in the store, time was paused such that they could spend as much time as needed to plan without decrementing their 10 min of field time. The provision of infinite in-store time was a design decision motivated by the need to allow teams the opportunity to engage in as much planning, externalization of information to create cognitive artifacts (cf. Fiore and Wiltshire, 2016), and discussion of resource allocation as possible to support the subsequent analysis of teamwork behaviors. While in the store, the team could use text-chat to collaboratively review their shared map (to both evaluate the information already recorded on the map as well as to add information such as planned operations and rally points), discuss how to spend their team budget to purchase tools (e.g., the color-coded bomb disposal tools, sensor tools for locating bombs, personal protective equipment, and more), review mission status and information, and vote to return to the field to continue bomb disposal operations.

At the start of each mission, teams were placed at a designated starting point in the bomb disposal field by the simulated testing environment and given 3 min for reconnaissance. During this phase, participants could search the area for bombs and use a limited supply of markers and communication beacons to share information about bomb locations and types on their shared mini-map. Each bomb presented unique challenges based on its parameters. Fuse timers varied, with some bombs set to detonate within minutes and others lasting nearly the full mission duration. Bombs also required specific disposal sequences, such as using a Red tool followed by two Blue tools (R-B-B), for safe deactivation. Failed disposals resulted in consequences, such as damage to nearby participants, and a subset of bombs triggered dynamically spreading fires. Some bombs were also “chained” and had to be deactivated in a specific order, with any deviation leading to immediate explosion. These parameters required teams to plan and coordinate carefully during reconnaissance and throughout the mission.

Teams completed their missions either by disposing of all bombs (either by successful disposal or a combination of explosions and disposals), running out of their 10 min of field time, or taking too much damage from bombs (note: when a given participant took too much damage they would be frozen in place and could be rescued by a team member; if all team members became frozen in place the mission ended in failure).

### 2.3 Taskwork and teamwork interdependencies

The main game mechanic that was leveraged to yoke taskwork and teamwork behaviors was the use of color-coded tools that were required for disposing of bombs across three dimensions of complexity: individual bomb sequence length, bomb sequence homogeneity, and cross-linking of bombs. Bombs could have sequences as low as one single step (e.g., just one tool needed) or as long as three steps and those steps could be completely homogeneous (e.g., three steps each requiring the use of the same type of tool) or heterogenous (e.g., each step requiring a different tool). There were three colors of bomb-disposal tools that participants could purchase from the in-game store interface: Red, Green, and Blue. The colors themselves were inconsequential, but participants could only carry any two different colors at a time (e.g., red and green, red and blue, or blue and green). Therefore, the distribution of colored tools across team members was an important resource for teams to manage because tools were consumed on use and bombs would begin an emergency 15-seconds-to-explosion countdown upon the application of a tool. To illustrate by example, consider a participant carrying eight red tools and six blue tools who encountered a 3-step bomb with the disposal sequence Red-Green-Blue. Although that participant could consume one of their red tools to begin the disposal, they would activate the emergency countdown but would not have a green tool that they could use to stop the impending explosion. Successfully disposing of that bomb would require assistance from a teammate who had a green tool because no one participant could ever carry all three types of tools. As an additional layer of complexity, the cross-linking of bombs required participants to carefully record and discuss the order in which some sets of bombs needed to be disposed of (e.g., a bomb sequence may be Bomb23, Bomb17, Bomb 44 requiring 23 to be disposed of before 17 and so forth).

### 2.4 Artificial social intelligences

Two ASIs were developed for this experiment. RITA (Rita, 2023) was developed by the Dynamic Object Language Labs in collaboration with the Massachusetts Institute of Technology. ATLAS (ATLAS, 2023) was developed by the Advanced Agent Robotics Technology Lab at Carnegie Mellon University. Because our focus is on understanding the impact of the human-ASI interactions on team outcomes and perceptions, details of the inner workings of the two ASI are beyond the scope of this paper. There are, however, a number of features of the agents that the reader should be familiar with to contextualize the nature of the advisors and their interactions with their human teammates. Generally, the advisors were designed to monitor the team’s behaviors (e.g., their movements, disposal actions, and communications) and to provide advice and interventions targeted at teamwork, not taskwork. The distinction between taskwork and teamwork here is important because it highlights the ASIST program’s goal of advancing AI that is not focused only on accomplishing tasks to advance teams’ goals, but to participate as active members in the team processes. As such, ASIST ASIs did not know anything about the mission environment that participants had not encountered and could not (or, rather, did not by design choice) solve the task for participants or tell them where they should go or what they should do when. Instead, RITA and ATLAS provided feedback on teams’ activities, identified gaps in shared knowledge, advised methods for maintaining awareness and offered backup assistance to teammates, and other teamwork focused interventions. We refer to RITA and ATLAS, collectively, as ASI advisors or just advisors.

### 2.5 Materials and measures

One of the core components of this article is examination of our individual and team profile models as they relate to various processes and outcomes in teams. The profiles are derived from six key components, constructed from both psychometric and psychographic measures, which assess an individual’s potential for taskwork and teamwork. The distinction between taskwork and teamwork was drawn from team theory, which differentiates between the competencies necessary for task completion and those required for effective collaboration. This distinction has been shown in our prior research to provide valuable insights into the behaviors and perceptions of teams working alongside ASI advisors (Bendell et al., 2024). The player profile model integrates five measures, with two focused on taskwork potential and three on teamwork potential. This approach aims to connect traditional methods of analyzing human behavior with contemporary techniques for designing artificial agents.

We define taskwork potential as the general abilities essential for carrying out tasks within virtual environments. This encompasses skills such as spatial navigation and memory for pathways, as well as the ease and confidence with which individuals complete tasks in gaming contexts. To evaluate spatial navigation skills, we employed the Santa Barbara Sense of Direction (SSOD) scale, a validated instrument recognized for its ability to predict success in navigating both physical and digital spaces (Hegarty et al., 2002). Additionally, the level of comfort and proficiency in completing tasks within gaming environments was gauged using the Video Game Experience Measure (Williams et al., 2023b; Williams, 2024), which specifically assesses gaming experience and competencies, with a focus on Minecraft and the USAR gamified scenarios.

The teamwork potential profile, the second dimension of the player profile, assessed broad competencies related to collaboration and interpersonal abilities. This dimension assessed individual’s abilities to understand others’ mental states and emotions, patterns of behavior in group settings, and engagement in collective activities and group dynamics. To measure those features of social intelligence, we used the Reading the Mind in the Eyes Test (Baron-Cohen et al., 2001). This is a validated instrument originally developed to identify subtle Theory of Mind deficits in adults with high-functioning autism, but also applicable to neurotypical individuals in making mental state judgments. Dispositions toward interaction behaviors were measured using the Sociable Dominance scale (Kalma et al., 1993). This is a validated tool that predicts social interaction behaviors, such as the likelihood of individuals with high sociable dominance to actively reason about their teammates and communicate directly in their interactions. Lastly, collective engagement and group behavior tendencies were assessed through the Psychological Collectivism scale (Jackson et al., 2006). This is a validated measure that examines attitudes toward group work, including personal tendencies when working with groups, awareness and engagement with group wellbeing, and adherence to the structures and systems agreed upon by the group.

To define participants’ profiles, individuals were assessed as having either high or low potential in both dimensions (taskwork and teamwork) based on their performance in the relevant assessments. Specifically, a person was classified as having high potential in a given category if they scored above the median on at least two of the measures employed for a given dimension. Conversely, if they scored low on two measures, they were considered to have low potential in that area. This classification system resulted in four distinct profile groups: low taskwork - low teamwork, high taskwork - low teamwork, low taskwork - high teamwork, and high taskwork - high teamwork potential.

Team level profiles were derived by aggregating the individual profiles of team members through a similar type of modal analysis as was used for defining each participants’ profile. In other words, the most frequently occurring taskwork and teamwork profiles among the team members determined the team’s overall classification. This approach allowed us to assign teams to categories of either high or low taskwork potential and high or low teamwork potential, mirroring the classification method used for individual players. For example, if a team was composed of one member with high taskwork but low teamwork, another with low taskwork and low teamwork, and a third with high taskwork and high teamwork, the team would be collectively classified as high taskwork (since two out of three members were categorized as such) and low teamwork (as two out of three members fell into this category).

### 2.6 Team and ASI advisor evaluations

Upon completing a mission with their team and ASI advisor, participants were asked to fill out a set of surveys designed to assess their perceptions of their team and their advisor. These survey items were each rated on a seven-point Likert scale ranging from “strongly disagree” to “strongly agree.” Team surveys included measures of participants' impressions of their team's collaboration and outcomes (e.g., satisfaction with score, collaborative effort, planning processes, desire to work together in the future, and general belief in their teams’ ability to perform), their team efficacy (e.g., work ethic, ability to overcome problems, plan a successful strategy, maintain positivity, succeed completely and with time to spare, to coordinate knowledge, and to coordinate to dispose of bombs efficiently), and their team process, for example, how well they share information (Mathieu et al., 2020).

Finally, team member also responded to five queries regarding their perceptions of their ASI advisor: “The advisor’s recommendations improved our team score”, “The advisor’s recommendations improved our team coordination”, “I felt comfortable depending on the advisor”, “I understand why the advisor made its recommendations”, “I think the AI Advisor is trustworthy”. Additionally, participants were asked to “please elaborate on your ratings of your AI advisor.”

## 3 Results

### 3.1 Study sample

Participants in this study were remotely located individuals who joined an online testbed environment, forming teams of three based on a first-come, first-served system. Consequently, an individual could participate multiple times, resulting in teams consisting of entirely new teammates, a mix of familiar and new teammates, or entirely previous teammates. Accordingly, the number of unique participants was not directly associated with the number of unique trials or teams that were formed during the experiment. The final dataset included 1,095 completed experimental trials with 276 unique individuals participating in various team configurations. The sample primarily consisted of young males (233 males, 41 females, two not reporting) with an average age of 20.1 years (ranging from 18 to 40 years); the nature of this sample should be considered when interpreting the results of the analyses presented here and the extent to which these findings are transferrable to different team compositions, including gender and age representation. With regards to the number of experimental trials completed by participants, the maximum number of trials completed by any one participant was 131, with a sample mean of 16.4 trials due to many participants completing only a single trial.

Although it is important to acknowledge the characteristics of individual participants, this article focuses on teams and the application of the taskwork and teamwork profiling method to assess and predict team performance when ASI is a member. The following analyses provide a detailed overview of the 1,095 teams that undertook the bomb disposal missions in the simulated testbed environment. Given the possibility of individuals participating multiple times, teams could consist of multiple experienced players or all novice players. To account for this, we report in our supplementary materials the total number of trials completed by the team members up to their participation together, as well as the maximum and minimum number of trials completed by individual team members for each mission (Supplementary Table 3).

Two important take-aways from our supplementary tables that the reader should keep in mind when considering the results of our analyses are the relative distributions of participant teams across the taskwork and teamwork profile dimensions, and the range and deviation of the number of trials across teams and team members. The distribution of teams across the profile categories was uneven; however, that was expected considering that the team level profiles arise from the individuals put together by the team formation system, which was essentially random and not in any way driven by individuals’ profiles. The most represented category in this team sample was low taskwork, low teamwork profiles (446 teams), followed by low taskwork, high teamwork (353 teams), then high taskwork, low teamwork (204 teams), and the least represented was high taskwork, high teamwork (92 teams). The second takeaway was that some teams were formed of relatively veteran members (having participated in the simulated bomb disposal missions up to 131 times) and others of entirely novice team members (having participated only once), see Supplementary Table 3. That is important to keep in mind when considering our pattern of findings, and we explore the possible implications of these varied team composition in the context of both the profiles and experience in later sections.

### 3.2 Correlational analyses

To investigate the relationships between teams’ profiles and their perceptions of their team processes as well as their evaluations of their ASI advisors, we first examined the intercorrelations between the outcome variables related to the two sets (Table 1). The first set of outcomes, team perceptions, was captured by five dependent variables: average team process rating (Team Process; Mathieu et al., 2000), average team satisfaction with their final score (Team Satisfaction Score), average team satisfaction with their planning behaviors (Team Satisfaction Planning), average team efficacy regarding strategy (Team Efficacy Strategy), and average team efficacy regarding knowledge coordination (Team Efficacy Knowledge Coord). The second set of outcomes, advisor evaluations (see Table 2), was captured by four dependent variables: average response to “The advisor’s recommendations improved our team score” (Advisor Eval Improved Score), average response to “The advisor’s recommendations improved our team coordination” (Advisor Eval Improved Coord), average response to “I felt comfortable depending on the advisor” (Advisor Eval Comfortable Depend On), and average response to “I think the AI Advisor is trustworthy” (Advisor Eval Trustworthy).

**TABLE 1:** Mediation analysis for the effect of Teamwork Potential and Taskwork Potential on team perceptions as mediated by Team Score.

		Direct effect	Indirect effect (through team score)	Total effect	Percent mediated (by team score)	Additional variance accounted for
Team Process	Teamwork Potential	0.3153	0.1674	0.4827	34.68%	39.09%
	Taskwork Potential	0.1478	0.2953	0.4431	66.6%	39.05%
Team Satisfaction rating Score	Teamwork Potential	0.09076	0.15861	0.24937	63.6%	29.27%
	Taskwork Potential	0.1430	0.2718	0.4147	65.5%	27.59%
Team Satisfaction rating Team Planning	Teamwork Potential	0.218	0.182	0.400	45.5%	39.13%
	Taskwork Potential	0.0987	0.3196	0.4183	76.4%	38.72%
Team Efficacy rating Team Strategy	Teamwork Potential	0.295	0.188	0.483	38.9%	40.41%
	Taskwork Potential	0.235	0.325	0.560	58.1%	38.98%
Team Efficacy rating Knowledge Coordination	Teamwork Potential	0.3378	0.1664	0.5042	33.0%	36.33%
	Taskwork Potential	0.230	0.290	0.520	55.8%	35.47%

**TABLE 2:** Mediation Analysis for the effect of Teamwork and Taskwork potential on advisor perceptions as mediated by Team Score.

		Direct effect	Indirect effect (through team score)	Total effect	Percent mediated (by team score)	Additional variance accounted for
Advisor evaluation Improved Score	Teamwork Potential	0.3820	0.0335	0.4154	8.0%	0.57%
	Taskwork Potential	0.4968	0.0471	0.5439	8.7%	0.36%
Advisor evaluation Improved Coordination	Teamwork Potential	0.4167	0.0011	0.4178	0.27%	<0.01%
	Taskwork Potential	0.3263	0.0010	0.3273	0.30%	<0.01%
Advisor evaluation Comfortable Depending On	Teamwork Potential	0.5106	0.0436	0.5541	7.9%	1.1%
	Taskwork Potential	0.4484	0.0720	0.5204	13.8%	0.98%
Advisor evaluation Was Trustworthy	Teamwork Potential	0.5813	0.0458	0.6271	7.3%	1.3%
	Taskwork Potential	0.5026	0.0758	0.5784	13.1%	1.2%

For the team perception variables, we anticipated that team members’ average responses could be highly correlated if teams tended to report general pleasure or displeasure uniformly. Each of the team perception outcome variables were highly correlated with each other such that teams that reported positive perceptions did so across all items and conversely those reporting negative perceptions responded accordingly to each item. These relationships do not imply that participants (and, by extension, teams) responded without deviation or nuance, but they do suggest that inspection of these outcomes should begin at the multivariate level. Similarly, correlational inspection of the second variable set, advisor evaluations, was conducted to address the assumption that team members may have responded generally positively or negatively across items. The four advisor evaluation variables exhibited high intercorrelation and therefore should be examined at the multivariate level, Supplementary Table 4 for the correlation analyses between Team Perception and Advisor Evaluation variables.

### 3.3 Mediation analyses: Influence of team score

Our expectation was that teams’ profile dimensions would predict their evaluations of their teams and of their advisors, but that the teams’ actual performance (i.e., score) could mediate their perceptions such that those teams that performed well would be biased towards more positive perceptions of their team and advisor. To examine the effects of the taskwork and teamwork profile dimensions on teams’ perceptions of their processes and performance as mediated by team score, we conducted a set of percentile bootstrapped mediation analyses (employing R library “mediation”; 5,000 resamples). The mediation analysis revealed significant effects of teamwork and taskwork potential on various team-related outcomes, with team score serving as a mediator such that teams that achieved better scores reported more positive perceptions (see Table 1). Overall, the mediation effects indicate that while team score significantly mediates the relationship between teamwork/taskwork potential and various team outcomes, the taskwork and teamwork profile dimensions also directly impact perceptions. Notably, teams higher in either profile dimension (or both dimensions) rated their teams more positively, regardless of score.

A similar set of mediation analyses conducted to investigate the possible mediating effect of team score on advisor evaluations did not reveal significant mediation by score. These analyses showed that unlike the relationship with team perceptions, team score did not notably impact teams’ ratings of their advisors (see Table 2).

The first takeaway from the above mediation analyses was that, as expected, team score significantly influenced teams’ perceptions of their process, their score, their satisfaction with their teaming behaviors, and their teamwork efficacy. Teams that performed better, accurately perceived the quality of their performance and their awareness seems to have increased their satisfaction and efficacy ratings notably.

The second takeaway was that team score did not seem to influence teams’ perceptions of their ASI advisor. This finding is particularly important because it suggests that the performance of the team (ostensibly, including the ASI advisor as a team member) did not change the way that the human team members viewed their advisor. Nonetheless, it is possible that human team members simply viewed their ASI advisor as separate entities (i.e., not part of the team) and therefore not responsible for the team’s performance or processes. Alternatively, it could be that they did view their ASI as an integral part of the team, but still did apply to them the standards of judgment that applied to their human compatriots.

### 3.4 Team profiles: Multivariate analyses of variance

We next conducted two separate two-way MANOVAs to test the relationships between teams’ two profile dimensions (taskwork potential, teamwork potential) and the two sets of constructs: team perceptions and advisor evaluations. The statistical analysis revealed significant effects within both the Team Perceptions Variable Cluster and the Advisor Evaluations Variable Cluster. For the Team Perceptions Variable Cluster, the analysis indicated a significant effect for Teamwork Potential, F (5, 1,024) = 23.432, p < 0.001, with Wilk’s Λ = 0.897, as well as for Taskwork Potential, F (5, 1,024) = 11.057, p < 0.001, with Wilk’s Λ = 0.949. However, the interaction between Teamwork and Taskwork was not statistically significant, F (5, 1,024) = 1.787, p = 0.113, with Wilk’s Λ = 0.991. Similarly, for the Advisor Evaluations Variable Cluster, there was a significant effect observed for Teamwork Potential, F (4, 1,025) = 16.788, p < 0.001, with Wilk’s Λ = 0.939, and for Taskwork Potential, F (4, 1,025) = 10.251, p < 0.001, with Wilk’s Λ = 0.962. The interaction between Teamwork and Taskwork was also significant in this cluster, F (4, 1,025) = 7.135, p < 0.001, with Wilk’s Λ = 0.973. The statistical significance of the MANOVAs indicates that the profile dimensions were able to distinguish differences in the participants perceptions. We next more closely examine those effects and the influence of the taskwork and teamwork traits.

### 3.5 Follow-on analyses of variance

#### 3.5.1 Team perceptions

**FIGURE 1:**
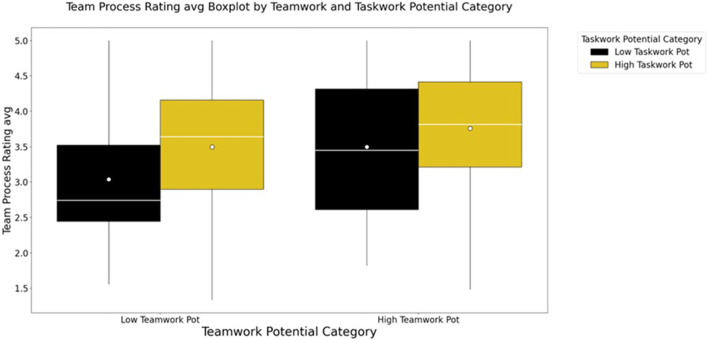
Team Process Average Ratings across Teamwork and Taskwork profile groups demonstrate that teams high in both dimensions report notably more positive perceptions of their teams than those teams high in only one dimension. All three groups of teams that were high in at least one dimension of the team profiles reported more positive perceptions than those teams low in both.

Closer inspection of the relationships between the team profile dimensions and the two sets of outcome variables, team perceptions and advisor evaluations, was conducted via follow-on two-way analyses of variance with Bonferroni corrections. Results indicate no interaction between the two profiles dimensions at the univariate level for any of the team perceptions variables. Teamwork potential and taskwork potential had separate, significant effects on each of the team perceptions outcome variables and were associated with small to moderate sized effects (see Table 3; Figure 1) shows that pattern as reflected by teams’ ratings of their team process. Generally, profiles distinguished ratings such that teams high in at least one dimension report notably more positive perceptions of their teams than those teams low in both dimensions.

**TABLE 3:** Two-way ANOVA results for Teamwork Potential, Taskwork Potential, and their Interaction on Team Perceptions.

	Teamwork potential	Taskwork potential	Interaction
Team Process Ratings (average)	F(1,1028) = 56.156 *p* < 0.001 ƞ^2^ = 0.050	F(1,1028) = 28.914 *p* < 0.001 ƞ^2^ = 0.026	F(1,1028) = 2.203 *p* = 0.138 ƞ^2^ = 0.002
Team Satisfaction Rating: Score	F(1,1028) = 7.526 *p* = 0.006 ƞ^2^ = 0.007	F(1,1028) = 28.006 *p* < 0.001 ƞ^2^ = 0.026	F(1,1028) = 2.094 *p* = 0.148 ƞ^2^ = 0.002
Team Satisfaction Rating: Planning	F(1,1028) = 29.175 *p* < 0.001 ƞ^2^ = 0.0270	F(1,1028) = 23.268 *p* < 0.001 ƞ^2^ = 0.0215	F(1,1028) = 1.663 *p* = 0.197 ƞ^2^ = 0.0015
Team Efficacy Rating: Strategy	F(1,1028) = 40.1839 *p* < 0.001 ƞ^2^ = 0.0360	F(1,1028) = 44.8431 *p* < 0.001 ƞ^2^ = 0.0403	F(1,1028) = 0.1937 *p* = 0.6599 ƞ^2^ = 0.0002
Team Efficacy Rating: Knowledge Coordination	F(1,1028) = 54.5409 *p* < 0.001 ƞ^2^ = 0.0484	F(1,1028) = 41.6303 *p* < 0.001 ƞ^2^ = 0.0370	F(1,1028) = 2.2452 *p* = 0.1343 ƞ^2^ = 0.0020

Post-hoc tests with Bonferroni correction were conducted to further investigate the differences between the profile dimension groups. The results indicate that both taskwork potential and teamwork potential have an effect on teams’ perceptions of their processes, their satisfaction with their performance, and their efficacy regarding teaming behaviors such that greater potential is associated with more positive perceptions (see Supplementary Table 6).

Although some *post hoc* comparisons indicated significant differences between the cross-potential groups (e.g., High Teamwork Low Taskwork, Low Taskwork High Teamwork) and the extreme-potential groups (i.e., High Teamwork High Taskwork, Low Teamwork Low Taskwork), no consistent pattern suggested that either teamwork potential or taskwork potential alone primarily drove the more positive responses. Instead, it seems that it is more important that a team be high in potential on at least one of the profile dimensions for them to report more positive perceptions of their team, and that being high potential in both dimensions is associated with the most positive perceptions (see Supplementary Figures S1–3).

#### 3.5.2 Advisor evaluations

Follow-on two-way ANOVAs were conducted to investigate the relationships between the two team profile dimensions and each of the advisor evaluation outcome variables. The pattern of results reflects the multivariate outcome above, indicating that there was some evidence of an interaction between the taskwork and teamwork potential dimensions such that teams higher in both reported more positive evaluations of their advisors. Notably, that interaction manifested significantly for only two of the outcome variables: Advisor Improved Team Coordination, and Advisor Improved Team Score (see Table 4). The main effect of taskwork potential was more consistent with evidence of its impact being demonstrated across three of the four outcome variables (excluding Advisor Improved Team Coordination), and a main effect of teamwork potential was found to be significant for all outcome variables.

**TABLE 4:** Two-way ANOVA results for Teamwork Potential, Taskwork Potential, and their Interaction on Advisor Evaluations.

	Teamwork potential	Taskwork potential	Interaction
ASI Improved Team Coordination Ratings	F(1,1028) = 18.7818 *p* < 0.001ƞ^2^ = 0.0177	F(1,1028) = 5.7998 *p* = 0.0162 ƞ^2^ = 0.0055	F(1,1028) = 10.4805 *p* = 0.0012 ƞ^2^ = 0.0099
ASI Improved Team Score Ratings	F(1,1028) = 11.6635 *p* < 0.001 ƞ^2^ = 0.0109	F(1,1028) = 19.3917 *p* < 0.001 ƞ^2^ = 0.0181	F(1,1028) = 12.2928 *p* < 0.001 ƞ^2^ = 0.0115
ASI Dependable Ratings	F(1,1028) = 29.4859 *p* < 0.001 ƞ^2^ = 0.0273	F(1,1028) = 16.5809 *p* < 0.001 ƞ^2^ = 0.0154	F(1,1028) = 4.8977 *p* = 0.0271 ƞ^2^ = 0.0045
ASI Trustworthy Ratings	F(1,1028) = 43.6577 *p* < 0.001 ƞ^2^ = 0.0399	F(1,1028) = 22.9113 *p* < 0.001 ƞ^2^ = 0.0209	F(1,1028) = 0.0245 *p* = 0.8757 ƞ^2^ = 0.00

**FIGURE 2:**
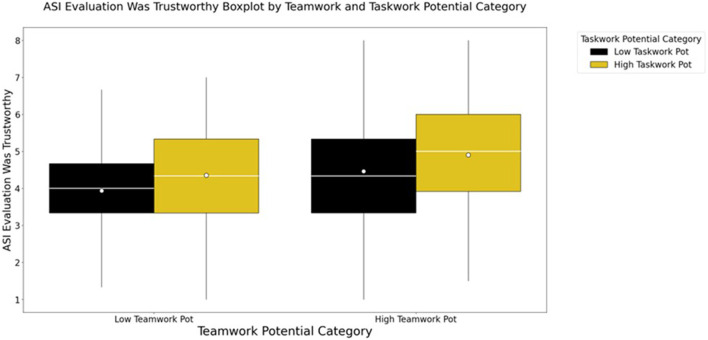
ASI Evaluation: Trustworthy Rating by Teamwork and Taskwork Potential profile groups demonstrate that teams high in both dimensions report notably more positive perceptions of their advisors than those teams high in only one dimension. Additionally, all three groups of teams that were high in at least one dimension of the team profiles reported more positive perceptions than those teams low in both.

Post-hoc analyses with Bonferroni correction revealed that differences between groups were partly driven by teams being high in at least one profile dimension: teams that were low in both taskwork and teamwork potential were associated with relatively more negative evaluations of their advisors. Significant differences between high and low groupings of both teamwork and taskwork potential were found, showing that teams that were high in both taskwork and teamwork potential (see Figure 2) or high in at least one dimension rated their advisors as notably more helpful than teams that were low potential in both dimensions (Supplementary Table 7; Supplementary Figures S4–7).

#### 3.5.3 Repeat teams

To explore the possible impacts of the number of times that a given team engaged in repeat play (i.e., had already completed bomb disposal missions), we split completed team trials into three groups: trials associated with the first time a team ever worked together (First Time), the second through the 10th time that a team worked together (2–10), and trials completed by teams that had worked together eleven or more times (11+). This allowed us to form two relatively equal groups of First Time (518 trials) and early teaming (2–10; 476 trials) trials in addition to a subset of trials completed by more veteran teams (11+; 101 trials). Similar to the previous exploration, we first conducted a one-way ANOVA to determine if the amount that a team had worked together impacted their score outcomes. Results of that analysis showed that there was a significant effect of team repeat play on score (F(2,1091) = 9.153, p < 0.001, ƞ2 = 0.017); however, it was not the case that more veteran teams outperformed more novice teams. First Time trials and 11+ trials were associated with similarly high scores (Mean Difference = −43.095, p = 0.645) whereas trials completed by the 2 to 10 group were associated with relatively lower scores compared to First Time (Mean Difference = 72.467, p = 0.001) and 11+ (Mean Difference = 115.562, p = 0.003).

Examining the interaction between the amount that a team had worked together and their two profile dimensions, we tested first the relationship between those three factors and the team perception variable set (five total) and next the advisor evaluation variable set (four total). Both analyses demonstrated that there were significant interactions between the three factors such that the amount that a team had worked together impacted the way that the teamwork and taskwork potential profiles manifested their effects on teams’ perceptions of themselves as well as their advisors. However, inspection of *post hoc* analyses revealed that the interactions were entirely driven by the previously determined effect of team potential (e.g., high potential groups outperform low potential groups) and the effect of team repeat trial count (e.g., group 2 to 10 underperformed relative to First Time and 11+) and did not evidence any new insight provided by the more complex model.

## 4 Discussion

Teaming is an important aspect of modern work, and the increasingly advanced capabilities of artificial agents have the potential to augment not only teams’ abilities to complete their taskwork but also to improve their teamwork behaviors. A necessary component of AI for supporting teamwork interactions will be the development of social intelligence founded on a form of artificial theory of mind that can allow agents to understand and make inferences about their human teammates. We suggest that the challenge of developing and integrating socially intelligent AI is a fundamentally interdisciplinary problem, that will require both technical developments to create ASI as well as teams research to identify how and when those agents contribute to effective teamwork or detract from it. One of the overarching goals of this line of research is to determine how ASI systems can best integrate into, and support, teams across a wide range of contexts and compositions. This involves not only designing systems that are effective for high-potential teams who are likely to perceive them positively but also developing interventions and strategies to support teams that may not naturally integrate ASI into their processes. Understanding how individual differences and inherent team traits influence these perceptions and usage tendencies is critical to creating ASI systems that can adapt to and benefit all types of teams.

This study was conducted to evaluate the impacts of ASI advisors interacting with virtually instanced teams completing simulated bomb disposal operations. As noted, this is one of the few human-AI teaming experiments conducted with functioning AI architectures. In this way we are able to assess how actual ASI, interacting with humans, is perceived by teammates, and how that relates to performance. Specifically, the testbed environment was designed to impose taskwork demands while ensuring opportunities for teamwork and collaboration advice to improve team effectiveness. The focus of the analyses reported above was primarily on the perceptions that teams formed of themselves as well as specifically of their assigned advisor. These factors are especially important for the near future of teaming with ASI, as artificial agents can support taskwork capabilities, but their capacity as teammates depends greatly on acceptance and integration into human teams.

The goal of the ASIST efforts was not to design better tools for humans to leverage, but to design partners that humans can rely on and interact with as they would any other good teammate. To that end, the ASI assisting with the bomb disposal missions were able to observe the actions of the teams as they conducted their disposal missions and to provide advice and insight to support their team’s process. Although the ASI in this study did not have large impacts on teams’ performance overall, they did demonstrate some positive impacts especially for teams that were high in taskwork and teamwork potential. Exploring the factors impacting team performance and collaborative behaviors was out of scope for this manuscript, but it is relevant to note here that high potential teams particularly seemed to benefit from suggestions that their ASI advisors made regarding the appropriate distribution of taskwork resources. Those team-related potential categorizations build upon our previous work to identify traits that may impact individuals’ perceptions of teammates both human and artificial (Bendell et al., 2024).

Regarding performance, those teams that were high in taskwork and teamwork potential seemed to respond more effectively to input related to balancing workload across team members. It is possible that although high potential teams were relatively more prepared to analyze the demands of the mission, they were predisposed to focus on individually executing taskwork and therefore benefited from the ASI’s prompts to consider collaborative effort and balancing workloads. Here, we focused primarily on aggregate measures of team perceptions; however, future work should closely examine the human-ASI interactions at the level of specific teams as well as particular collaboration events. The publicly available ASIST study four dataset provides researchers with a unique opportunity to analyze human-agent team communication events, taskwork actions, movements, decision-making outcomes, and more, as these data were captured at a high temporal resolution, with observations recorded every few hundred milliseconds. This rich and detailed dataset holds significant potential for future research to delve into the temporal dynamics of human-agent interactions and team processes over time.

Reflecting on perceptions of the ASI, our findings suggest that they may not have been perceived as equal or contributing teammates. A notable distinction was apparent in our mediation analyses, which indicated that teams’ objective performance (reflected by their team score) strongly influenced their team perceptions but not their perceptions of their ASI advisors. Teams that performed better perceived themselves more positively, but not their ASI advisors. The relationship between performance and perceptions may be interpreted in different ways. On the one hand, it could indicate that team members did not consider their advisors as an integral part of their success. On the other hand, it is possible they did not hold them responsible for their failure. It is also possible that it was some combination of both amounting to the essential exclusion of the advisors as a member of the team. Future research is needed to explore why this occurs, as it addresses the crucial question of how artificial intelligence can be considered a teammate rather than a tool. It is important to note that this study did not find clear performance benefits for teams working with ASI advisors compared to what might be expected from human-only teams. This aligns with prior findings that ASI, if not adequately imbued with social intelligence, or allowed to fully leverage its computational and informational capabilities, can hinder rather than help team performance (Bendell et al., 2024). While the potential for ASI to significantly augment teamwork is widely recognized, particularly in leveraging their speed, memory, and information access, realizing these benefits requires a careful understanding of how ASI systems impact team dynamics. As socially intelligent AI continues to advance, researchers must prioritize the development of systems that integrate seamlessly into teams as effective teammates rather than functioning solely as tools. This includes addressing both technical and social dimensions of team integration to ensure that ASI systems are able to contribute meaningfully to team success.

One crucial factor that must be addressed for near-future teaming will be the interface that supports interactions between humans and socially intelligent AI teammates. As described, due to constraints in real-time natural language processing, our artificial intelligence was limited in how it could communicate with human teammates and how they could respond. Specifically, the ASI used a pop-up text box to provide advice, and the human teammates were limited in how they could respond, with multiple choice options provided. Depending on when this was provided, it could be viewed as intrusive as well as given when it was difficult for the humans to understand or appreciate their advisor’s input while continuing to execute their mission. The communication systems were designed to balance the human-subjects experimentation needs with the technical capacities of the ASI. This, though, provides additional avenues for research. Specifically, not only can modes of communication be varied and tested, this can be examined in the context of our profiling method. However, it is important to keep in mind when considering the ASIST study four data that the communication modalities employed were fundamentally limiting and could have been confusing or difficult for the human teammates to use. It could be that those high in task potential are more amenable to pop-up interventions when compared to those with less potential for these types of tasks. Further, as ASI gets more sophisticated, it can provide an intervention in a way most appropriate to the mission context. For example, given that workload is increasingly assessable in real time (Wiltshire et al., 2024), in the near future ASI can monitor workload and interventions with different modalities can be tested. Overcoming difficult problems such as natural language processing to allow human team members to communicate smoothly and in their preferred format with all of their teammates, especially artificial ones, and testing which method is most effective, will be vital for supporting human-ASI teaming.

Another challenging factor for the ASI advisors was the need to adjust the level and nature of their strategic advice to the experience level of the team members with whom they were working. On the one hand, our profiling technique does provide a surrogate for shared mental models, by helping the ASI to understand more generic competencies indicative to potential in the team and task setting. On the other hand, the ASI should be able to also track more idiosyncratic behaviors of teams based upon monitoring their missions over time and learning from these. As currently designed, the ASI operating in this study was not able to track or integrate knowledge regarding individual participants’ previous missions (if they had completed any), or to employ that information to adjust the focus and type of feedback they provided. As such, future research can examine how ASI can learn from repeated experience with their human teammates in a way that more naturally aligns with learning in human-human teams. This adaptability will be especially important for supporting teams that may struggle with integrating ASI systems. By combining real-time assessment of taskwork and teamwork traits with insights from repeated interactions, ASI systems can adjust their communication styles and interventions to better align with the needs of their human teammates. This approach could provide a more equitable distribution of ASI support, ensuring that lower-potential teams also experience meaningful benefits.

A related issue is how repeat participants may have used the testbed environment. It is possible that some viewed early missions as a way to test the system and for learning task mechanics and strategies. Perhaps the task was complex enough to impose challenges and encourage more complicated team processes that advanced teams might learn to execute. When that is coupled with ASI that has a limited range of interventions, this results in an artificial intelligence that provides relatively simplistic strategies and teamwork advice to “veteran” teams as well as beginners. This interpretation is somewhat supported by our finding that the learning curve for this experiment was non-linear, and that teams with moderate experience performed worse than those with none or those with a great deal of experience. This provides a path forward for more advanced ASI that is able to acquire and retain knowledge of their teammates and their prior experiences, so that they can integrate as a team member at the level their counterparts need rather than providing input that is seemingly out-of-touch with the expertise or abilities of their team members.

Perceptions of ASI were not, however, uniform across all types of team members. As was found in our prior research (Bendell et al., 2024), taskwork and teamwork traits did predict differences and possible biases in participants’ views of both their teams and advisors. Focusing first on the advisors, we found the two profile dimensions interacted such that teams high in both taskwork and teamwork potential rated their AI advisors as more dependable, trustworthy, and effective in improving team coordination and score. In other words, though each potential (taskwork and teamwork) individually improved the perception of the ASI, their combination significantly increased teams’ appreciation for the advisor’s role. This could indicate that high potential teams are better at integrating ASI advice into their processes, that they are more appreciative of external guidance, or that the traits of the team members predispose them to positive biases when evaluating their advisors. The interaction of the taskwork and teamwork dimensions was most notable for perceptions of the advisors’ impacts on team coordination and team score. Keeping in mind that actual objective outcomes did not influence advisor perceptions (as shown by the mediation analyses), this may indicate that high potential teams are more likely to view ASI as members of the team who are responsible for team outcomes. It may also be that teams higher in both dimensions may have had an easier time working with the interfaces that supported interactions with the ASI as well as team knowledge externalization, planning, and resource management thereby leading those teams to report relatively more positive perceptions.

Regarding team perceptions, we found that teams featuring members with relatively higher taskwork potential or teamwork potential reported more positive views of their teams. Although that impact was significantly mediated by the actual performance of teams, the effect of higher potential across either profile dimension was evident and associated with positive perceptions of team process, satisfaction with team behaviors, and team’s self-efficacy. However, there was no interaction between the taskwork and teamwork profile dimension. This may suggest that, while both contributed to positive views, there was no additive value between the two that distinguished high taskwork, high teamwork teams from others as was seen for advisor perceptions. That is to say, teams that were higher in taskwork potential may have felt more positively about their teaming experience because they had an easier time performing the tasks regardless of their collaboration, and teams that were higher in teamwork potential may have reported more positively based on their engagement in team processes even if they struggled with taskwork.

As we suggest, team profiles have the potential to provide AI with more immediate insights on their human teammates. In some ways this can circumvent learning via interaction by accelerating acquisition of teammate relevant knowledge. As such, this contributes to AI’s shared cognition, potentially improving their effectiveness in supporting teamwork. But this knowledge will need to be tailored to the varied potentials of human team members. For example, tailoring ASI systems to also support teams with lower taskwork and teamwork potential will require identifying and addressing the specific needs of each team, as not all low-potential teams are alike. While some teams may benefit from more foundational task-oriented guidance, others might require strategies focused on improving collaboration and team cohesion. Our findings suggest that high-potential teams perceived their ASI advisors more positively, but these perceptions were not directly tied to performance outcomes, highlighting the role of individual differences and team composition in shaping attitudes toward ASI. To better understand how ASI can support lower-potential teams, studies could intentionally construct teams with lower taskwork or teamwork potential. This approach would allow researchers to systematically investigate how specific interventions, communication strategies, or feedback styles influence different types of low-potential teams. For instance, ASI systems might incorporate real-time profiling techniques to adapt their feedback pacing, style, or level of detail based on the unique traits of a team. Addressing these questions is critical for developing ASI systems that not only excel with high-functioning teams but also meaningfully contribute to the success of diverse team compositions.

Building on our findings, there is a clear need to further investigate how perceptions and team dynamics evolve over time, particularly within the subset of teams that completed multiple missions together. Key questions include whether team members’ perceptions of their ASI advisors or teammates shift with repeated interactions, whether their in-mission behaviors become more structured or consistent, and how evolving expertise influences their engagement with the ASI. Additionally, comparisons between teams’ progressions (for example, a given team’s 12th or 20th mission) must account for contextual factors such as the randomized nature of team assignments and the first-in-first-out lobby system used in this study. While the current dataset’s randomized structure introduces variability, it also allows for the exploration of broader patterns across diverse team compositions. Careful consideration of these factors will be essential for deriving meaningful insights into the temporal development of human-ASI collaboration and its implications for both task effectiveness and teamwork processes.

Our findings related to the taskwork and teamwork profiles offer insights that can guide future research on optimizing human-ASI teaming. The different perceptions evidenced across team profile categories points to the importance of testing ASI systems in a variety of team contexts, ideally those in which the profiles of team members are manipulated to differ significantly. Future studies could explore how ASI can be designed to cater to different compositions of teams, potentially developing more flexible ASI that can adjust behavior and advice based on the team’s traits. As our understanding of relevant traits develops and evidence gathers to support their impact on aspects of taskwork and teamwork success, ASI would also benefit from the ability to assess the taskwork and teamwork potential of the teams in real-time. By doing so, ASI advisors could tailor their interventions to the specific needs and capabilities of a team, providing more nuanced and strategic guidance to high-potential teams and perhaps offering more foundational support to teams with lower potential. It is possible that such adaptability would enhance the perceived value of ASI and increase the likelihood that they are viewed as an integral part of the team rather than as an external or redundant presence.

Future research on human-AI teaming should examine the generalizability of team profiles and ASI across varied tasks. For instance, one line of inquiry could focus on developing ASI tailored to specific task domains to holistically evaluate how team traits influence teaming outcomes. It remains unclear which team traits, if any, generalize across tasks to affect acceptance, trust, and willingness to work with ASI. Different teaming scenarios will likely emphasize different traits, providing ASI with context-specific guidance on how to best support their teammates. Our prior study using an Urban Search and Rescue task environment (Bendell et al., 2024) revealed similar patterns to the current Bomb Disposal task, both of which encourage collaboration by linking better performance to successful execution of interdependent tasks. However, many aspects of team-ASI relationships across these domains remain unexplored.

Another important avenue involves more granular analyses of team coordination. Current analyses have primarily focused on aggregate team outcomes, leaving questions about how ASI interactions influence in-mission behaviors unanswered. Patterns of communication, near-term behavior adjustments in response to ASI input, impacts on knowledge sharing, and the maintenance of team mental models have yet to be closely examined. Leveraging the high-temporal-resolution data captured in this study offers a unique opportunity for time-series analyses to assess behavior changes within missions. Such analyses could provide valuable insights into the temporal dynamics of ASI-to-human exchanges, human-to-human communications, and team movements, uncovering how ASI interventions shape coordination, decision-making, and task execution. These findings would inform the design of future ASI systems that dynamically adapt to evolving team needs in mission-critical operations.

Additionally, while this study focused on artificial social intelligence designed to support teamwork, future research should also explore how ASI can integrate teamwork facilitation with task-oriented interventions. ASI will need to balance these roles effectively, requiring capabilities to provide both types of support. Research should develop and test ASI systems that can independently or jointly address task-oriented and team-oriented contributions, allowing researchers to evaluate the unique and combined impacts of these interventions. This could involve designing ASI capable of both roles with the flexibility to activate or deactivate each function as needed, enabling more precise assessments of their contributions to team outcomes.

Overall, our findings emphasize the importance of continued research into the human factors that shape ASI acceptance and effectiveness as both are essential for developing AI teammates that can genuinely enhance the collaborative capabilities of human teams. Further, our work highlights the need for ASI systems that can dynamically assess and adapt to the specific taskwork and teamwork profiles of their human counterparts. In this way it builds on a long line of research on teams and aspects of team mental models (Cannon-Bowers et al., 1993) to test how profiling can be used as a surrogate for ASI understanding of their human teammates. By tailoring their support to the unique needs and strengths of each team, ASI can make a more significant contribution to both team performance and the overall teaming experience.

The ability for ASI to assess and respond to team traits will be an essential component of an effective artificial theory of mind. This capability enables these systems to function as effective teammates rather than just tools. By creating ASI that can interpret and act upon the implications of human traits within specific task contexts, we can move closer to developing AI that not only augments human abilities but also enriches the collaborative process. Achieving this goal will require interdisciplinary efforts that combine technical advancements with comprehensive teams research, guiding the design of ASI systems that enhance both the efficiency and the experience of teamwork across various domains.

## Data Availability

The datasets presented in this study can be found in online repositories. The names of the repository/repositories and accession number(s) can be found below: https://dataverse.asu.edu/dataset.xhtml?persistentId=doi:10.48349/ASU/ZO6XVR.

## Author disclaimer

The views and conclusions contained in this document are those of the authors and should not be interpreted as representing the official policies, either expressed or implied, of DARPA, the Army Research Office, or the U.S. Government. The Government is authorized to reproduce and distribute reprints for Government purposes notwithstanding any copyright notation herein.
