# Supplementary material for: Artificial social intelligence in teamwork: how team traits influence human-AI dynamics in complex tasks
Source: Front Robot AI. 2025 Feb 17;12:1487883. doi: 10.3389/frobt.2025.1487883 (PMC11873349; doi:10.3389/frobt.2025.1487883)
Supplement: Supplementary file 1 [file DataSheet1.docx]

Supplementary Table 1 Team scores on taskwork-teamwork profiling measures.

|  | Team Psychological Collectivism (0-5) | Team Social Dominance (0-5) | Team Reading the Mind in the Eyes (0-10) | Team Spatial Ability (0-6) | Team Video Game Experience (0-100) |
| --- | --- | --- | --- | --- | --- |
| Mode | 3.4 | 3.2 | 6.0 | 4.0 | 71.4 |
| Median | 3.5 | 3.2 | 5.3 | 4.2 | 66.3 |
| Mean | 3.5 | 3.2 | 5.2 | 4.3 | 66.3 |
| Std. Deviation | 0.38 | 0.36 | 2.01 | 0.5 | 7.6 |
| Minimum | 2.2 | 2.1 | 0.5 | 2.3 | 35.6 |
| Maximum | 4.7 | 4.5 | 10.0 | 6.0 | 84.2 |

Supplementary Table 2 Descriptive statistics for the distribution of teams across the dimensions of taskwork and teamwork potential

|  | Low Taskwork | High Taskwork |
| --- | --- | --- |
| Low Teamwork | N = 446 | N = 92 |
| High Teamwork | N = 353 | N = 204 |

Supplementary Table 3 Rates of participation across teams.

|  | Team Number of Trials Sum (Same 3 Team Members) | Player Number of Trials (Max of Team Members) | Player Number of Trials (Min of Team Members) |
| --- | --- | --- | --- |
| Mode | 156.000 | 67.000 | 6.000 |
| Median | 117.000 | 59.000 | 19.000 |
| Mean | 123.816 | 57.056 | 26.646 |
| Std. Deviation | 73.718 | 32.293 | 20.537 |
| Minimum | 3.000 | 1.000 | 1.000 |
| Maximum | 333.000 | 131.000 | 85.000 |

Supplementary Table 4 Correlation table for Team Perception and Advisor Evaluation Variables.

| Variable Cluster | Variable 1 | Variable 2 | Pearson's r | *p* value |
| --- | --- | --- | --- | --- |
| Team Perceptions Cluster | Team Process Rating avg | Team Satisfaction Rating Score | 0.766 | < .001 |
|  | Team Process Rating avg | Team Satisfaction Rating Team Planning | 0.891 | < .001 |
|  | Team Process Rating avg | Team Efficacy Rating Team Strategy | 0.905 | < .001 |
|  | Team Process Rating avg | Team Efficacy Rating Knowledge Coordination | 0.871 | < .001 |
|  | Team Satisfaction Rating Score | Team Satisfaction Rating Team Planning | 0.752 | < .001 |
|  | Team Satisfaction Rating Score | Team Efficacy Rating Team Strategy | 0.761 | < .001 |
|  | Team Satisfaction Rating Score | Team Efficacy Rating Knowledge Coordination | 0.691 | < .001 |
|  | Team Satisfaction Rating Team Planning | Team Efficacy Rating Team Strategy | 0.860 | < .001 |
|  | Team Satisfaction Rating Team Planning | Team Efficacy Rating Knowledge Coordination | 0.845 | < .001 |
|  | Team Efficacy Rating Team Strategy | Team Efficacy Rating Knowledge Coordination | 0.868 | < .001 |
| ASI Evaluation Cluster | ASI Evaluation: Improved Score | ASI Evaluation: Improved Coordination | 0.868 | < .001 |
|  | ASI Evaluation: Improved Score | ASI Evaluation: Comfortable Depending On | 0.835 | < .001 |
|  | ASI Evaluation: Improved Score | ASI Evaluation: Was Trustworthy | 0.700 | < .001 |
|  | ASI Evaluation: Improved Coordination | ASI Evaluation: Comfortable Depending On | 0.865 | < .001 |
|  | ASI Evaluation: Improved Coordination | ASI Evaluation: Was Trustworthy | 0.734 | < .001 |
|  | ASI Evaluation: Comfortable Depending On | ASI Evaluation: Was Trustworthy | 0.826 | < .001 |

Supplementary Table 5 Two-way ANOVA results for Teamwork Potential, Taskwork Potential and their Interaction on Team Perceptions and Advisor Evaluations.

|  | Teamwork Potential | Taskwork Potential | Teamwork-Taskwork Interaction |
| --- | --- | --- | --- |
| Team Perceptions Variable Cluster | F(5,1024) = 23.432  *p* < .001  Wilk’s Λ = 0.897 | F(5,1024) = 11.057  *p* < .001  Wilk’s Λ = 0.949 | F(5,1024) = 1.787  *p* = .113  Wilk’s Λ = 0.991 |
| Advisor Evaluations Variable Cluster | F(4,1025) = 16.788  *p* < .001  Wilk’s Λ = .939 | F(4,1025) = 10.251  *p* < .001  Wilk’s Λ = .962 | F(4,1025) = 7.135  *p* < .001  Wilk’s Λ = .973 |

Supplementary Table 6 Bonferroni corrected Post-hoc results for Teamwork Potential and Taskwork Potential on Team Perceptions

|  | Group 1 | Group 2 | Mean diff | *p* |
| --- | --- | --- | --- | --- |
| Team Process Perceptions | High Teamwork Pot | Low Teamwork Pot | -0.481 | < .001 |
|  | High Taskwork Pot | Low Taskwork Pot | -0.444 | < .001 |
| Team Score Satisfaction | High Teamwork Pot | Low Teamwork Pot | -0.247 | < .001 |
|  | High Taskwork Pot | Low Taskwork Pot | -0.416 | < .001 |
| Team Planning Satisfaction | High Teamwork Pot | Low Teamwork Pot | -0.398 | < .001 |
|  | High Taskwork Pot | Low Taskwork Pot | -0.420 | < .001 |
| Team Strategy Efficacy | High Teamwork Pot | Low Teamwork Pot | -0.480 | < .001 |
|  | High Taskwork Pot | Low Taskwork Pot | -0.562 | < .001 |
| Team Knowledge Coordination Efficacy | High Teamwork Pot | Low Teamwork Pot | -0.501 | < .001 |
|  | High Taskwork Pot | Low Taskwork Pot | -0.522 | < .001 |


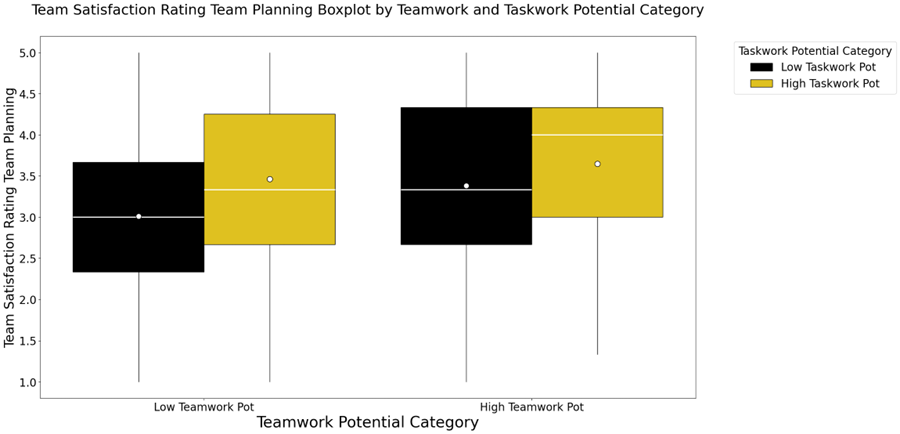


Supplementary Figure 1. Team Rating: Satisfaction with Team Plan by Teamwork and Taskwork profile groups demonstrate that teams high in at least one dimension report notably more positive perceptions of their teams than those teams low in both dimensions.


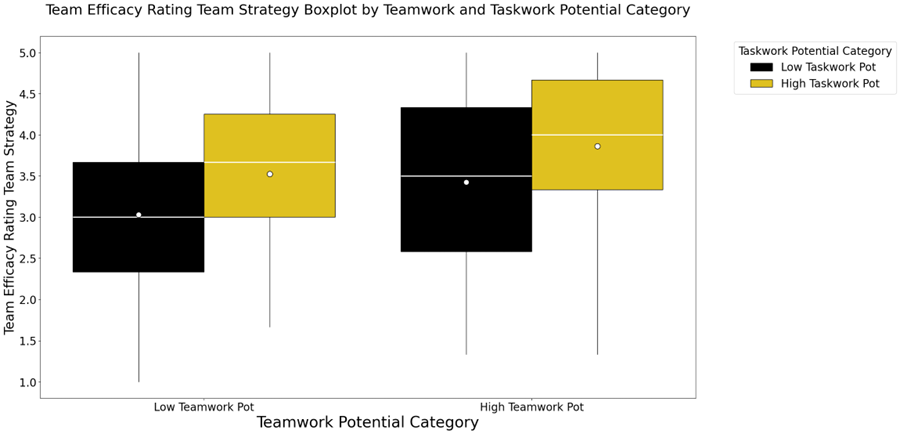


Supplementary Figure 2. Team Efficacy Rating: Strategy by Teamwork and Taskwork profile groups demonstrate that teams high in both dimensions report notably more positive perceptions of their teams than those teams high in only one dimension. All three groups of teams that were high in at least one dimension of the team profiles reported more positive perceptions than those teams low in both.


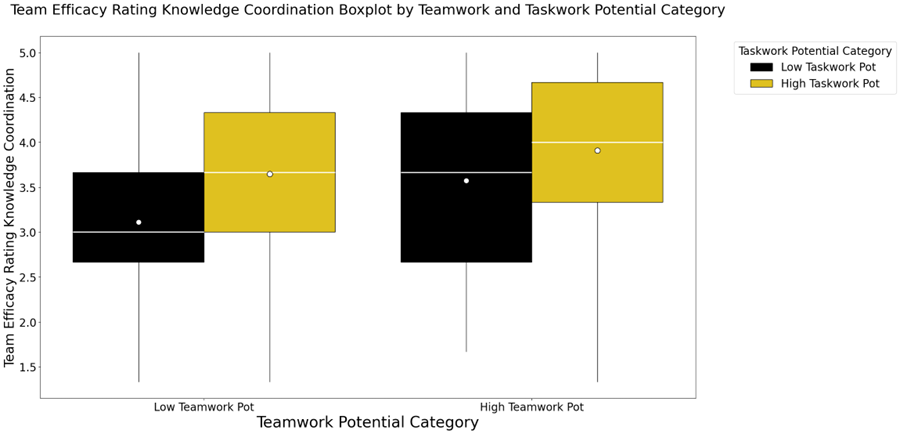


Supplementary Figure 3. Team Efficacy Rating: Knowledge Coordination by Teamwork and Taskwork profile groups demonstrate that teams high in at least one dimension report notably more positive perceptions of their teams than those teams low in both dimensions.

Supplementary Table 7 Bonferroni correction differences between High and Low groupings of both Teamwork and Taskwork potential.

|  | Group 1 | Group 2 | Mean diff | *p*-adj |
| --- | --- | --- | --- | --- |
| Advisor Improved Team Coordination | High Teamwork Potential | Low Teamwork Potential | -0.415 | < .001 |
|  | High Taskwork Potential | Low Taskwork Potential | -0.329 | < .001 |
| Advisor Improved Team Score | High Teamwork Potential | Low Teamwork Potential | -0.412 | < .001 |
|  | High Taskwork Potential | Low Taskwork Potential | -0.546 | < .001 |
| Advisor Comfortable Depending On | High Teamwork Potential | Low Teamwork Potential | -0.551 | < .001 |
|  | High Taskwork Potential | Low Taskwork Potential | -0.522 | < .001 |
| Advisor Was Trustworthy | High Teamwork Potential | Low Teamwork Potential | -0.624 | < .001 |
|  | High Taskwork Potential | Low Taskwork Potential | -0.580 | < .001 |

**
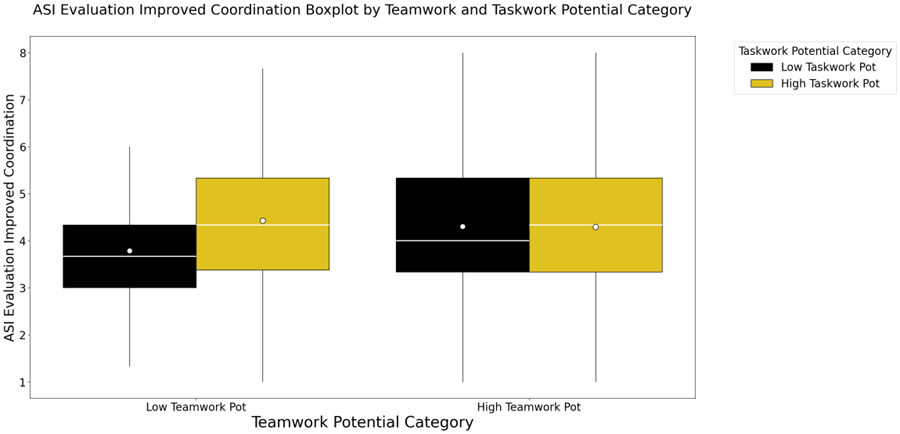
**

Supplementary Figure 4. ASI Evaluation: Improved Coordination by Teamwork and Taskwork Potential profile groups demonstrate that teams high in at least one dimension report notably more positive perceptions of their advisors than those teams low in both dimensions.


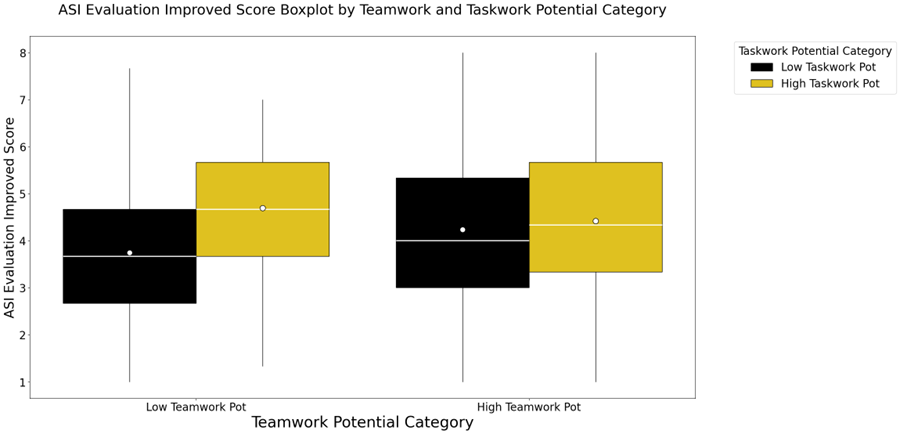


Supplementary Figure 5. ASI Evaluation: Improved Score by Teamwork and Taskwork Potential profile groups demonstrate that teams high in at least one dimension report notably more positive perceptions of their advisors than those teams low in both dimensions.


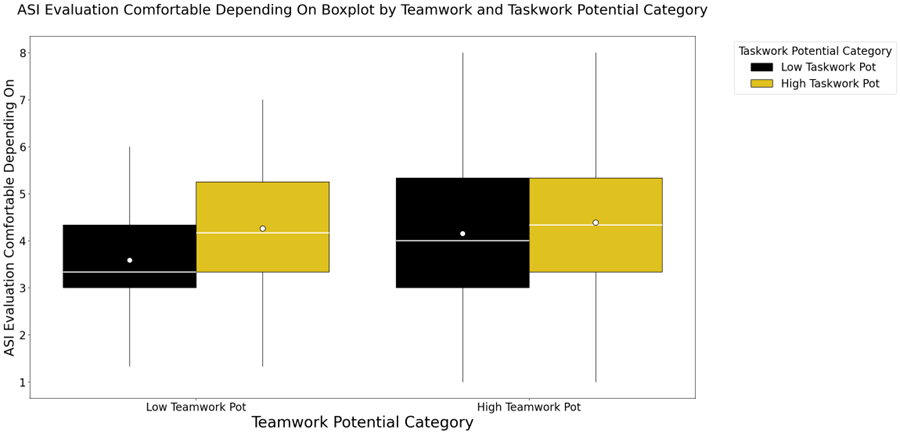


Supplementary Figure 6. ASI Evaluation: Dependable Rating by Teamwork and Taskwork Potential profile groups demonstrate that teams high in at least one dimension report notably more positive perceptions of their advisors than those teams low in both dimensions.
